# Supplementary material for: The Influence of the Exclusion of Central Necrosis on [18F]FDG PET Radiomic Analysis
Source: Diagnostics (Basel). 2021 Jul 19;11(7):1296. doi: 10.3390/diagnostics11071296 (PMC8304274; doi:10.3390/diagnostics11071296)
Supplement: Supplementary file 1 [file diagnostics-11-01296-s001.zip › Supplementals diagnostics/supplemental 1.pdf]

Supplementary File 1: Image Biomarker Standardisation Initiative Reporting Guidelines

Patient

	NSCLC	PPGL
1. Volumes of interest	NSCLC showing central necrosis based on semi-automatic threshold-based tumour delineation	PPGL showing central necrosis based on semi-automatic threshold-based tumour delineation
2. Patient preparation<br>a. Patient instructions<br>b. Drugs<br>c. Equipment	Patients fasted for at least 6 hours before imaging and were orally hydrated with 500 mL of water.	
3. Radioactive tracer:<br>a. Tracer<br>b. Administration method<br>c. Injected activity<br>d. Uptake time prior to acquisition<br>e. Competing substances	Dynamic [^{18}F]FDG PET was performed for one bed-position with the tumour centrally located in the field of view.<br>Directly after the start of the acquisition, a standardized infusion of 3.45 MBq of [^{18}F]FDG per kilogram of body weight was started. The intravenous administration was injected in an intracubital vein contralateral to the tumor by using a standardized injection protocol (8.0 mL at 0.2 mL/sec) with a remote-controlled pump, followed by a saline flush (40 mL at 8.0 mL/sec).<br>Serum glucose levels were below 8.0 mmol/L.	PET acquisition was started 60 (55-75) minutes after intravenous administration of [^{18}F]FDG in an intracubital vein, using a non-linear dosage (D) regimen:<br>- <95 kg body weight (BW): D = 1.7*BW (4 min per bed position)<br>- >95 kg BW: D = 0.017778*W^2 (4 min per bed position)<br>- 3 min/bed position: D*1.333<br>- 2 min/bed position: D*2<br>- Maximum: 555 MBq, minimum: 20 MBq<br>Serum glucose levels were below 8.0 mmol/L.
4. Contrast agent	NA	
5. Comorbidities	Patients with DM were allowed as long as serum glucose was below 8.0 mmol/L and no short-acting insulins were given 4 hours prior to injection of FDG.	Patients with DM were allowed as long as serum glucose was below 8.0 mmol/L and no short-acting insulins were given 4 hours prior to injection of FDG.<br>In case of high serum levels of catecholamines

	Patients with active pulmonary infections were excluded.	it is known that brown adipose tissue (BAT) uptake can be strongly increased. As BAT can be present in the perirenal fossa, it could interfere with delineation of the pheochromocytoma. Patients with pheochromocytomas and high BAT uptake in the perirenal fossa were excluded.

Acquisition

	NSCLC	PPGL
6. Acquisition protocol	After performing low-dose breath-hold CT, free-breathing PET acquisition was performed for 60 minutes.	A low-dose CT was performed, followed by a static PET scan, from the base of the skull to the mid-thigh, approximately 60 minutes post-injection.
7. Scanner type	Siemens Biograph Duo and Siemens Biograph 40 mCT (Siemens Healthineers, Erlangen, Germany)	Siemens Biograph 40 mCT (Siemens Healthineers, Erlangen, Germany)
8. Imaging modality	PET/CT	
9. Static/dynamic scans<br>a. Static/dynamic<br>b. Acquisition time per frame<br>c. Temporal modelling technique	Dynamic scans<br>20 x 5 s<br>5 x 10 s<br>10 x 15 s<br>10 x 30 s<br>16 x 75 s<br>8 x 150 s<br>1 x 600 s<br>Only the final time frame (50–60 min p.i.) was used in this analysis. Temporal modelling was not used for this analysis.	Static scans
10. Scanner calibration	All PET/CT scanners were regularly cross-calibrated with the dose calibrator of the hospital pharmacy and the dose calibrator of the pharmacy that delivers the FDG according to EARL-guidelines and guidelines for quality control of the Dutch Association of Nuclear Medicine (NVNG).	
11. Patient instructions	Free-breathing PET scans were acquired with the patient instructed not to move and positioned in restraining/supportive devices.	

12. Anatomical motion correction	No anatomical motion correction is performed, due to the hybrid nature of PET-CT it is assumed that the position of PET and CT with respect to the scanner's coordinate system stays constant.	
13. Scan duration	60 minutes. The final time frame (50–60 min p.i.) was used in this analysis.	3 or 4 min/bed position, dose adjusted accordingly
14. Tube voltage ldCT	130 kVp	130 kVp
15. Tube current ldCT	40 mA	40 mA
16. Time-of-flight	Biograph Duo: no<br>Biograph mCT: yes	Yes

Reconstruction

	NSCLC		PPGL
	Biograph Duo	Biograph 40 mCT	Biograph 40 mCT
28. In plane resolution	2.65×2.65 mm²	1.59×1.59 mm²	3.18×3.18 mm²
29. Image slice thickness	3.38 mm	2.03 mm	3.00 mm
30. Image slice spacing	3.38 mm	2.03 mm	3.00 mm
32. Reconstruction method<br>a. Method<br>b. Number of iterations<br>c. Number of subsets	Ordered-subsets expectation maximization with 4 iterations per 16 subsets	High-definition ordered-subsets expectation maximation with 3 iterations per 21 subsets	
33. Point spread function modelling	NA	Point spread function based time of flight	
34. Image corrections<br>a. Attenuation correction<br>b. Other corrections	AC: based on low-dose CT<br>Correction for scatter, randoms, normalization, dead time and physical decay was applied.		

Image processing – data conversion

	NSCLC		PPGL
	Biograph Duo	Biograph 40 mCT	Biograph 40 mCT
38. SUV normalisation	NA		
40. Other data conversions	NA		

Image processing – post acquisition processing

	NSCLC		PPGL
	Biograph Duo	Biograph 40 mCT	Biograph 40 mCT
41. Anti-aliasing	NA		

42. Noise suppression	NA		
43. Post-reconstruction smoothing filter	5 mm Gaussian	3 mm Gaussian	8 mm Gaussian
46. Intensity normalisation	NA		
47. Other post-acquisition processing methods	NA		

Segmentation

	NSCLC	PPGL
48. Segmentation method<br>a. Method<br>b. Number of experts, expertise, consensus strategies<br>c. Settings<br>d. Images	VOIs were delineated semi-automatically using 3DSlicer (www.slicer.org) and in-house built software implemented in Python 3.7 (Python Software Foundation, Wilmington, Delaware).<br><br>$VOI_{vital\text{-}tumour}$: delineated using a 41% isocontour method of the SUV_{peak}, obtained using a sphere of 12 mm diameter, corrected for local background, boxing was applied to exclude surrounding [^{18}F]FDG-avid tissues.<br>$VOI_{gross\text{-}tumour}$: manual addition of the volumes of central necrosis to $VOI_{vital\text{-}tumour}$, using low-dose CT as a visual reference.<br><br>VOIs were delineated by WN (PhD candidate PET radiomics with 3 years of experience) and supervised by DV (nuclear medicine physician with 12 years of experience)	
49. Conversion to mask	NA	

Image processing – image interpolation

	NSCLC	PPGL
50. Interpolation algorithm<br>a. Algorithm<br>b. Interpolation grid<br>c. Dimensions<br>d. Extrapolation	Trilinear interpolation using MATLAB 2017b (Mathworks, Natick, Massachusetts), grids were aligned by centre, values were rounded to the nearest integer.	NA
51. Interpolated voxel dimensions	3.38×3.38×3.38 mm^3	NA

Image processing – ROI interpolation and re-segmentation

	NSCLC	PPGL
53. Interpolation algorithm	NA, VOIs were delineated on interpolated images	NA
54. Partially masked voxels	NA	NA
55. Re-segmentation methods	NA	NA

Image processing – discretisation

56. Discretisation method<br>a. Method<br>b. Number of bins/bin size<br>c. Lowest intensity first bin	Discretisation using a fixed bin size of 0.5 g/mL. Bin edges were equally spaced from 0 (e.g. 0-0.5, 0.5-1, etc) and the lowest grey value was discretized into the first bin.

Image processing – image transformation

57. Image filter	NA	NA

Image biomarker computation

58. Biomarker set	• First Order Statistics (18 features): 10th Percentile, 90th Percentile, Energy, Entropy, Interquartile range, Kurtosis, Maximum, Mean absolute deviation, Mean, Median, Minimum, Range, Robust mean absolute deviation, Root mean squared, Skewness, Total energy, Uniformity, Variance<br>• Shape based (14 features): Elongation, Flatness, Least axis length, Major axis length, Maximum 2D diameter column, Maximum 2D diameter row, Maximum 2D diameter slice, Maximum 3D diameter, Mesh volume, Minor axis length, Sphericity, Surface area, Surface volume ratio, Voxel volume<br>• Grey Level Cooccurrence Matrix (GLCM; 22 features): Autocorrelation, Joint Average, Cluster Prominence, Cluster Shade, Cluster Tendency, Contrast, Correlation, Difference Average, Difference Entropy, Difference Variance, Joint Energy (= Angular Second Moment), Joint Entropy, Informational Measure of Correlation 1, Informational Measure of Correlation 2, Inverse Difference Moment, Inverse Difference Moment Normalized, Inverse Difference, Inverse Difference Normalized, Inverse Variance, Maximum Probability (= Joint Maximum), Sum Entropy, Sum of Squares (=Joint Variance)<br>• Grey Level Run Length Matrix (GLRLM; 16 features): Short Run Emphasis, Long Run Emphasis, Grey Level Non-Uniformity, Grey Level Non-Uniformity Normalized, Run Length Non-Uniformity, Run Length Non-Uniformity Normalized, Run Percentage, Grey Level Variance, Run Variance, Run Entropy, Low Grey Level Run Emphasis, High Grey Level Run Emphasis, Short Run Low Grey Level Emphasis,

	Short Run High Grey Level Emphasis, Long Run Low Grey Level Emphasis, Long Run High Grey Level Emphasis<br>• Grey Level Size Zone Matrix (GLSZM; 16 features): Small Area Emphasis, Large Area Emphasis, Grey Level Non-Uniformity, Grey Level Non-Uniformity Normalized, Size-Zone Non-Uniformity, Size-Zone Non-Uniformity Normalized, Zone Percentage, Grey Level Variance, Zone Variance, Zone Entropy, Low Grey Level Zone Emphasis, High Grey Level Zone Emphasis, Small Area Low Grey Level Emphasis, Small Area High Grey Level Emphasis, Large Area Low Grey Area Emphasis, Large Area High Grey Level Emphasis<br>• Grey Level Dependence Matrix (GLDM; 14 features): Small Dependence Emphasis, Large Dependence Emphasis, Grey Level Non-Uniformity, Dependence Non-Uniformity, Dependence Non-Uniformity Normalized, Grey Level Variance, Dependence Variance, Dependence Entropy, Low Grey Level Emphasis, High Grey Level Emphasis, Small Dependence Low Grey Level Emphasis, Small Dependence High Grey Level Emphasis, Large Dependence Low Grey Level Emphasis, Large Dependence High Grey Level Emphasis<br>• Neighbouring Grey Tone Difference Matrix (NGTDM; 5 features): Coarseness, Contrast, Busyness, Complexity, Strength
59. IBSI compliance	Yes
60. Robustness	Not assessed
61. Software availability	PyRadiomics 3.0 in Python 3.7 (Python Software Foundation, Wilmington, Delaware)

Image biomarker computation – texture parameters

62. Texture matrix aggregation	GLCM and GLRLM: 3D: average; GLSZM, GLDM and NGTDM: 3D
63. Distance weighting	No weighting
64. Cooccurrence matrix symmetry	Symmetric
65. Cooccurrence matrix distance	Chebyshev distance of 1
66. Size zone matrix linkage distance	Chebyshev distance of 1
67. Distance zone matrix linkage distance	NA
68. Distance zone matrix distance norm	NA
69. Neighbouring grey tone difference matrix distance	Chebyshev distance of 1

70. Grey level dependence matrix distance	Chebyshev distance of 1
71. Grey level dependence matrix coarseness	0

Machine learning and radiomics analysis

72. Diagnostic and prognostic modelling<br>a. Objective<br>b. Population<br>c. Outcome<br>d. Predictors<br>e. Performance<br>f. Limitations	Compare predictive performances of radiomic models based on different delineation methods (adaptive threshold 41% SUV_{peak} ($VOI_{vital\text{-}tumour}$), central necrosis filled ($VOI_{gross\text{-}tumour}$) and combined)<br><br>31 patients with pheochromocytomas and paragangliomas, retrospectively included<br><br>Noradrenergic biochemical profile (yes: 13, no: 18)<br><br>Radiomic features as assessed redundancy filtering and factor analysis, 1 feature per 10 patients:<br>- $VOI_{vital\text{-}tumour}$: First order Minimum, Shape Surface Area, GLCM Informational Measure of Correlation 2<br>- $VOI_{gross\text{-}tumour}$: Shape Surface Area*, NGTDM Complexity, GLDM Dependence Entropy<br>- Combined: $VOI_{gross\text{-}tumour}$ GLCM Sum entropy, $VOI_{vital\text{-}tumour}$ Shape maximum 3D diameter, $VOI_{vital\text{-}tumour}$ Shape Surface Volume ratio<br><br>AUCs (95% CI):<br>- $VOI_{vital\text{-}tumour}$: 0.829 (0.677-0.981)<br>- $VOI_{gross\text{-}tumour}$: 0.803 (0.640-0.967)<br>- Combined: 0.791 (0.618-0.963)<br><br>Model was used for the comparison of predictive performance between delineation methods and was not developed and validated for predictive purposes.
73. Comparison with known factors	NA
74. Multicollinearity	Unsupervised dimension reduction using redundancy filtering (threshold = 0.9) and factor analysis was performed using FMradio (Factor Modeling for Radiomics Data).
75. Model availability	Model was used for the comparison of predictive performance between delineation methods and was not developed and validated for predictive purposes.
76. Data availability	The datasets generated during and/or analysed during the current study are available from the corresponding author on reasonable request.
