# Supplementary material for: The Influence of the Exclusion of Central Necrosis on [18F]FDG PET Radiomic Analysis
Source: Diagnostics (Basel). 2021 Jul 19;11(7):1296. doi: 10.3390/diagnostics11071296 (PMC8304274; doi:10.3390/diagnostics11071296)
Supplement: Supplementary file 1 [file diagnostics-11-01296-s001.zip › Supplementals diagnostics/supplementary table S1.pdf]

## Supplementary Table S1: features affected by delineation method concerning central necrosis

Features affected by the choice of delineation method ($VOI_{vital\text{-}tumour}$ vs $VOI_{gross\text{-}tumour}$) indicated with a check (✓) for the different cohorts and subgroups based on NTF and $SUV_{max}$. Per feature class, the number of affected features per cohort is indicated by a number. NSCLC: non-small cell lung carcinoma, PPGL: pheochromocytoma and paraganglioma, NTF: necrotic tumour fraction, $SUV_{max}$: maximum standardized uptake value, GLCM: grey level cooccurrence matrix, GLRLM: grey level run length matrix, GLSZM: grey level size zone matrix, GLDM: grey level dependence matrix, NGTDM: neighbouring grey tone difference matrix.

| | NSCLC (N=12) | PPGL (N=31) | All (N=43) | Small (N=14) | Medium (N=15) | Large (N=14) | Low $SUV_{max}$ (N=14 | Medium $SUV_{max}$ (N=15) | High $SUV_{max}$ (N=14) |
|---|---|---|---|---|---|---|---|---|---|
| First order (18) | 13 | 16 | 17 | 14 | 16 | 14 | 14 | 15 | 16 |
| 10th Percentile | ✓ | ✓ | ✓ | ✓ | ✓ | ✓ | ✓ | ✓ | ✓ |
| 90th Percentile | | ✓ | ✓ | ✓ | ✓ | ✓ | ✓ | ✓ | ✓ |
| Energy | ✓ | ✓ | ✓ | | ✓ | | | ✓ | ✓ |
| Entropy | ✓ | ✓ | ✓ | ✓ | ✓ | ✓ | ✓ | ✓ | ✓ |
| Interquartile range | | ✓ | ✓ | ✓ | ✓ | ✓ | ✓ | | ✓ |
| Kurtosis | | | ✓ | | | | | | |
| Maximum | | | | | | | | | |
| Mean absolute deviation | ✓ | ✓ | ✓ | ✓ | ✓ | ✓ | ✓ | ✓ | ✓ |
| Mean | ✓ | ✓ | ✓ | ✓ | ✓ | ✓ | ✓ | ✓ | ✓ |
| Median | ✓ | ✓ | ✓ | ✓ | ✓ | ✓ | ✓ | ✓ | ✓ |
| Minimum | ✓ | ✓ | ✓ | ✓ | ✓ | ✓ | ✓ | ✓ | ✓ |
| Range | ✓ | ✓ | ✓ | ✓ | ✓ | ✓ | ✓ | ✓ | ✓ |
| Robust mean absolute deviation | | ✓ | ✓ | ✓ | ✓ | ✓ | ✓ | ✓ | ✓ |
| Root mean squared | ✓ | ✓ | ✓ | ✓ | ✓ | ✓ | ✓ | ✓ | ✓ |
| Skewness | ✓ | ✓ | ✓ | ✓ | ✓ | ✓ | ✓ | ✓ | ✓ |
| Total energy | ✓ | ✓ | ✓ | | ✓ | | | ✓ | ✓ |
| Uniformity | ✓ | ✓ | ✓ | ✓ | ✓ | ✓ | ✓ | ✓ | ✓ |

| | | | | | | | | | |
|---|---|---|---|---|---|---|---|---|---|
| Variance | ✓ | ✓ | ✓ | ✓ | ✓ | ✓ | ✓ | ✓ | ✓ |
| Shape (14) | 7 | 7 | 11 | 5 | 8 | 10 | 4 | 5 | 4 |
| Elongation | | | ✓ | | | | | | |
| Flatness | | ✓ | ✓ | | ✓ | ✓ | | ✓ | |
| Least axis length | | ✓ | ✓ | | | ✓ | | | |
| Major axis length | ✓ | | | | ✓ | | | | |
| Maximum 2D diameter column | | | ✓ | | | ✓ | | | |
| Maximum 2D diameter row | | ✓ | ✓ | | | ✓ | | | |
| Maximum 2D diameter slice | | | ✓ | | | ✓ | | | |
| Maximum 3D diameter | | | ✓ | | | ✓ | | | |
| Mesh volume | ✓ | ✓ | ✓ | ✓ | ✓ | ✓ | ✓ | ✓ | ✓ |
| Minor axis length | ✓ | | | | ✓ | | | | |
| Sphericity | ✓ | ✓ | ✓ | ✓ | ✓ | ✓ | ✓ | ✓ | ✓ |
| Surface area | ✓ | | | ✓ | ✓ | | | | |
| Surface volume ratio | ✓ | ✓ | ✓ | ✓ | ✓ | ✓ | ✓ | ✓ | ✓ |
| Voxel volume | ✓ | ✓ | ✓ | ✓ | ✓ | ✓ | ✓ | ✓ | ✓ |
| GLCM (22) | 12 | 15 | 15 | 13 | 15 | 15 | 15 | 12 | 12 |
| Autocorrelation | ✓ | ✓ | ✓ | ✓ | ✓ | ✓ | ✓ | ✓ | ✓ |
| Joint average | ✓ | ✓ | ✓ | ✓ | ✓ | ✓ | ✓ | ✓ | ✓ |
| Cluster prominence | ✓ | ✓ | ✓ | ✓ | ✓ | ✓ | ✓ | ✓ | ✓ |
| Cluster shade | | ✓ | ✓ | ✓ | ✓ | ✓ | ✓ | | |
| Cluster tendency | ✓ | ✓ | ✓ | ✓ | ✓ | ✓ | ✓ | ✓ | ✓ |
| Contrast | | | | | | | | | |
| Correlation | ✓ | ✓ | ✓ | ✓ | ✓ | ✓ | ✓ | ✓ | ✓ |
| Difference average | | | | | | ✓ | | | |
| Difference entropy | | | | | | | | | |
| Difference variance | | | | | | | | | |

| | | | | | | | | | |
|---|---|---|---|---|---|---|---|---|---|
| Joint energy | | ✓ | ✓ | | ✓ | | ✓ | | |
| Joint entropy | ✓ | ✓ | ✓ | ✓ | ✓ | ✓ | ✓ | ✓ | ✓ |
| Informational measure correlation 1 | ✓ | ✓ | ✓ | ✓ | ✓ | ✓ | ✓ | ✓ | ✓ |
| Informational measure correlation 2 | ✓ | ✓ | ✓ | ✓ | ✓ | ✓ | ✓ | ✓ | ✓ |
| Inverse difference moment | | | | | | ✓ | | | |
| Inverse difference moment normalized | ✓ | ✓ | ✓ | ✓ | ✓ | ✓ | ✓ | ✓ | ✓ |
| Inverse difference | | | ✓ | | | | | | |
| Inverse difference normalized | ✓ | ✓ | ✓ | ✓ | ✓ | ✓ | ✓ | ✓ | ✓ |
| Inverse variance | | | | | | | | | |
| Maximum probability | | ✓ | | | ✓ | | ✓ | | |
| Sum entropy | ✓ | ✓ | ✓ | ✓ | ✓ | ✓ | ✓ | ✓ | ✓ |
| Sum squares | ✓ | ✓ | ✓ | ✓ | ✓ | ✓ | ✓ | ✓ | ✓ |
| GLRLM (16) | 12 | 15 | 15 | 12 | 14 | 14 | 9 | 13 | 15 |
| Grey level nonuniformity | | | | | | | | | |
| Grey level nonuniformity normalized | ✓ | ✓ | ✓ | ✓ | ✓ | ✓ | ✓ | ✓ | ✓ |
| Grey level variance | ✓ | ✓ | ✓ | ✓ | ✓ | ✓ | ✓ | ✓ | ✓ |
| High grey level run emphasis | ✓ | ✓ | ✓ | ✓ | ✓ | ✓ | ✓ | ✓ | ✓ |
| Long run emphasis | ✓ | ✓ | ✓ | | | | | ✓ | ✓ |
| Long run high grey level emphasis | ✓ | ✓ | ✓ | ✓ | ✓ | ✓ | ✓ | ✓ | ✓ |
| Long run low grey level emphasis | | ✓ | ✓ | | ✓ | ✓ | | ✓ | ✓ |
| Low grey level run emphasis | ✓ | ✓ | ✓ | ✓ | ✓ | ✓ | ✓ | ✓ | ✓ |

| | | | | | | | | | |
|---|---|---|---|---|---|---|---|---|---|
| Run entropy | ✓ | ✓ | ✓ | ✓ | ✓ | ✓ | ✓ | ✓ | ✓ |
| Run length nonuniformity | ✓ | ✓ | ✓ | ✓ | ✓ | ✓ | ✓ | ✓ | ✓ |
| Run length nonuniformity normalized | ✓ | ✓ | ✓ | ✓ | ✓ | ✓ | | ✓ | ✓ |
| Run percentage | | ✓ | ✓ | | ✓ | ✓ | | | ✓ |
| Run variance | | ✓ | ✓ | ✓ | ✓ | ✓ | | ✓ | ✓ |
| Short run emphasis | ✓ | ✓ | ✓ | ✓ | ✓ | ✓ | | | ✓ |
| Short run high grey level emphasis | ✓ | ✓ | ✓ | ✓ | ✓ | ✓ | ✓ | ✓ | ✓ |
| Short run low grey level emphasis | ✓ | ✓ | ✓ | ✓ | ✓ | ✓ | ✓ | ✓ | ✓ |
| GLSZM (16) | 11 | 13 | 12 | 12 | 15 | 9 | 7 | 11 | 12 |
| Grey level nonuniformity | | | | ✓ | ✓ | | | | |
| Grey level nonuniformity normalized | ✓ | ✓ | ✓ | ✓ | ✓ | ✓ | ✓ | ✓ | ✓ |
| Grey level variance | ✓ | ✓ | ✓ | ✓ | ✓ | ✓ | ✓ | ✓ | ✓ |
| High grey level zone emphasis | ✓ | ✓ | ✓ | ✓ | ✓ | ✓ | ✓ | ✓ | ✓ |
| Large area emphasis | | ✓ | ✓ | ✓ | ✓ | | | ✓ | |
| Large area high grey level emphasis | ✓ | ✓ | ✓ | ✓ | ✓ | ✓ | ✓ | ✓ | ✓ |
| Large area low grey level emphasis | | ✓ | | | ✓ | | | | |
| Low grey level zone emphasis | ✓ | ✓ | ✓ | ✓ | ✓ | ✓ | ✓ | ✓ | ✓ |
| Size zone nonuniformity | | | | | | | | | ✓ |
| Size zone nonuniformity normalized | ✓ | ✓ | ✓ | ✓ | ✓ | | | ✓ | ✓ |
| Small area emphasis | ✓ | | | | ✓ | | | | ✓ |

| | | | | | | | | | |
|---|---|---|---|---|---|---|---|---|---|
| Small area high grey level emphasis | ✓ | ✓ | ✓ | ✓ | ✓ | ✓ | ✓ | ✓ | ✓ |
| Small area low grey level emphasis | ✓ | ✓ | ✓ | ✓ | ✓ | ✓ | | ✓ | ✓ |
| Zone entropy | ✓ | ✓ | ✓ | ✓ | ✓ | ✓ | ✓ | ✓ | ✓ |
| Zone percentage | ✓ | ✓ | ✓ | | ✓ | ✓ | | ✓ | ✓ |
| Zone variance | | ✓ | ✓ | ✓ | ✓ | | | | |
| GLDM (14) | 9 | 13 | 12 | 10 | 11 | 11 | 8 | 9 | 10 |
| Dependence entropy | ✓ | ✓ | ✓ | ✓ | ✓ | ✓ | ✓ | ✓ | ✓ |
| Dependence nonuniformity | ✓ | ✓ | ✓ | ✓ | ✓ | ✓ | | ✓ | ✓ |
| Dependence nonuniformity normalized | | ✓ | ✓ | | ✓ | | | | ✓ |
| Dependence variance | | ✓ | ✓ | | | ✓ | | | |
| Grey level nonuniformity | | | | | | | | | |
| Grey level variance | ✓ | ✓ | ✓ | ✓ | ✓ | ✓ | ✓ | ✓ | ✓ |
| High grey level emphasis | ✓ | ✓ | ✓ | ✓ | ✓ | ✓ | ✓ | ✓ | ✓ |
| Large dependence emphasis | | ✓ | ✓ | ✓ | ✓ | ✓ | | | |
| Large dependence high grey level emphasis | ✓ | ✓ | ✓ | ✓ | ✓ | ✓ | ✓ | ✓ | ✓ |
| Large dependence low grey level emphasis | | ✓ | | | | | | | |
| Low grey level emphasis | ✓ | ✓ | ✓ | ✓ | ✓ | ✓ | ✓ | ✓ | ✓ |
| Small dependence emphasis | ✓ | ✓ | ✓ | ✓ | ✓ | ✓ | ✓ | ✓ | ✓ |
| Small dependence high grey level emphasis | ✓ | ✓ | ✓ | ✓ | ✓ | ✓ | ✓ | ✓ | ✓ |

| | | | | | | | | | |
|---|---|---|---|---|---|---|---|---|---|
| Small dependence low grey level emphasis | ✓ | ✓ | ✓ | ✓ | ✓ | ✓ | ✓ | ✓ | ✓ |
| NGTDM (5) | 4 | 4 | 4 | 5 | 5 | 2 | 2 | 4 | 4 |
| Busyness | ✓ | ✓ | ✓ | ✓ | ✓ | ✓ | ✓ | ✓ | ✓ |
| Coarseness | | | | ✓ | ✓ | | | | |
| Complexity | ✓ | ✓ | ✓ | ✓ | ✓ | ✓ | ✓ | ✓ | ✓ |
| Contrast | ✓ | ✓ | ✓ | ✓ | ✓ | | | ✓ | ✓ |
| Strength | ✓ | ✓ | ✓ | ✓ | ✓ | | | ✓ | ✓ |
| Total (105) | 83 | 86 | 68 | 71 | 84 | 75 | 59 | 69 | 73 |
