# Supplementary figures and images for: The Influence of the Exclusion of Central Necrosis on [18F]FDG PET Radiomic Analysis
Source: Diagnostics (Basel). 2021 Jul 19;11(7):1296. doi: 10.3390/diagnostics11071296 (PMC8304274; doi:10.3390/diagnostics11071296)

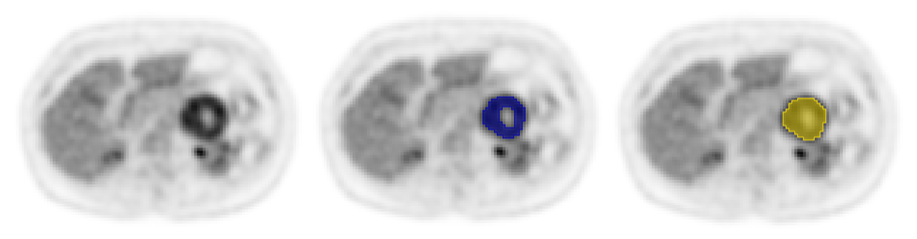

Supplement: Supplementary file 1 [file diagnostics-11-01296-s001.zip › Supplementals diagnostics/Fig1.tif]

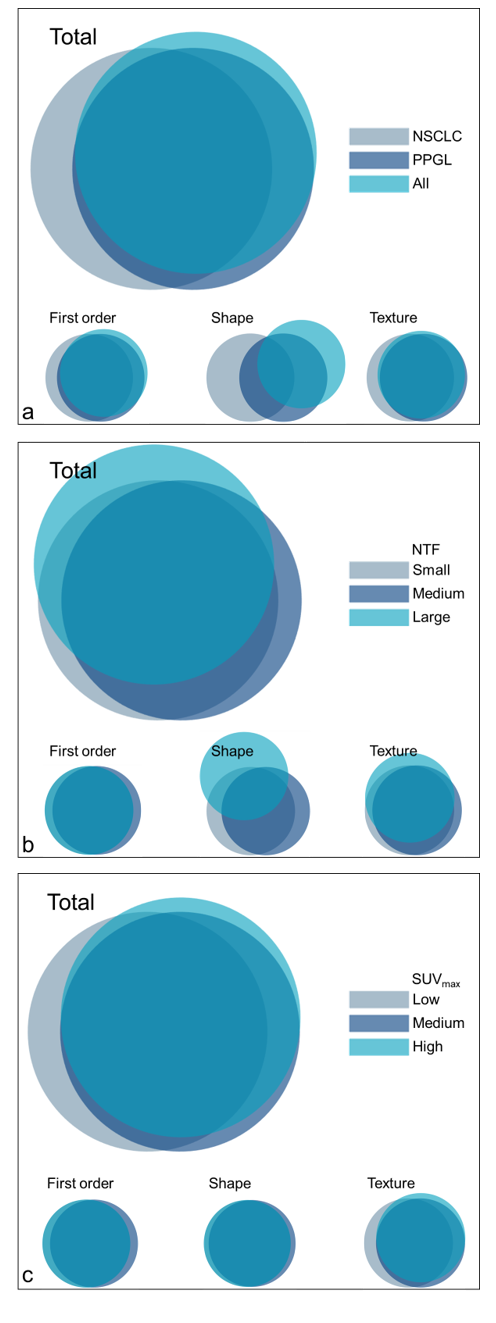

Supplement: Supplementary file 1 [file diagnostics-11-01296-s001.zip › Supplementals diagnostics/Fig2.tif]

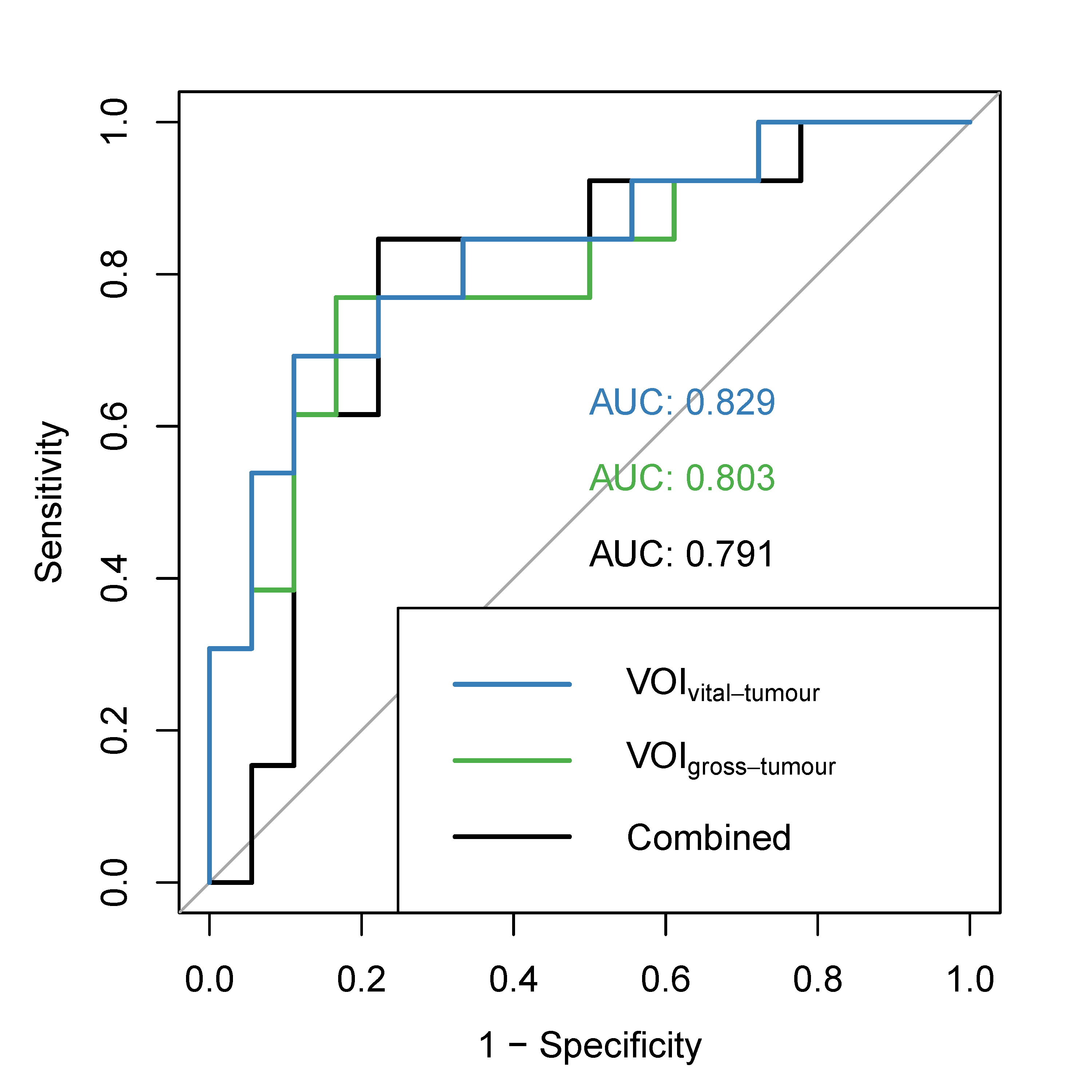

Supplement: Supplementary file 1 [file diagnostics-11-01296-s001.zip › Supplementals diagnostics/Fig3.tif]
